# Risk assessment of maize damage by wireworms (Coleoptera: Elateridae) as the first step in implementing IPM and in reducing the environmental impact of soil insecticides

**DOI:** 10.1007/s11356-016-7692-z

**Published:** 2016-10-06

**Authors:** L. Furlan, B. Contiero, F. Chiarini, M. Colauzzi, E. Sartori, I. Benvegnù, F. Fracasso, P. Giandon

**Affiliations:** 1 Veneto Agricoltura, Viale dell’Università, 14, 35020 Legnaro, PD Italy; 2 Dipartimento di Medicina Animale, Produzioni e Salute—Università degli Studi di Padova, Viale dell’Università, 16, 35020 Legnaro, PD Italy; 3 ARPAV Servizio Osservatorio Suolo e Bonifiche, Via S. Barbara, 5/a, 31100 Treviso, TV Italy

**Keywords:** *Agriotes brevis*, *Agriotes sordidus*, *Agriotes ustulatus*, Wireworms, Maize, Damage risk factors, Multifactorial model, Mutual-fund

## Abstract

A survey of maize fields was conducted in northeast Italy from 1986 to 2014, resulting in a dataset of 1296 records including information on wireworm damage to maize, plant-attacking species, agronomic characteristics, landscape and climate. Three wireworm species, *Agriotes brevis* Candeze, *A. sordidus* Illiger and *A. ustulatus* Schäller, were identified as the dominant pest species in maize fields. Over the 29-year period surveyed, no yield reduction was observed when wireworm plant damage was below 15 % of the stand. A preliminary univariate analysis of risk assessment was applied to identify the main factors influencing the occurrence of damage. A multifactorial model was then applied by using the significant factors identified. This model allowed the research to highlight the strongest factors and to analyse how the main factors together influenced damage risk. The strongest factors were: *A. brevis* as prevalent damaging species, soil organic matter content >5 %, rotation including meadows and/or double crops, *A. sordidus* as prevalent damaging species, and surrounding landscape mainly meadows, uncultivated grass and double crops. The multifactorial model also showed how the simultaneous occurrence of two or more of the aforementioned risk factors can conspicuously increase the risk of wireworm damage to maize crops, while the probability of damage to a field with no-risk factors is always low (<1 %). These results make it possible to draw risk maps to identify low-risk and high-risk areas, a first step in implementing bespoke IPM procedures in an attempt to reduce the impact of soil insecticides significantly.

## Introduction

The implementation of integrated pest management (IPM) strategies against wireworms has been extremely difficult due to the lack of available information on the key aspects of the species concerned (Furlan 2005). IPM strategies have not played a significant role in maize and other arable crops to date (Furlan and Kreutzweiser 2015) despite the strong negative impact of using soil insecticides (i.e., neonicotinoids) to control wireworms (van der Sluijs et al. 2015). EU Directive 2009/128/EC on the sustainable use of pesticides made it compulsory to implement IPM for all annual crops in Europe from January 2014. Therefore, accurate information about IPM strategies for annual crops is urgently needed, but this information must take into account that arable farming has few resources in terms of income, labour and technology. In order to implement IPM at low cost, it is important to establish the risk factors that cause an increase in wireworm population levels and the consequent damage. This research aimed to:

- find out the main entomological, agronomical and climatic factors that significantly increase the risk of wireworm damage;
- assess the most probable maize damage level in the presence of one or more risk factors; and
- establish the most effective procedure to implement IPM of wireworms in maize, considering the major risk factors that increase wireworm populations.

## Materials and methods

An extensive survey of maize fields was conducted in northeast Italy (area covered: 45.64 °N, 12.96 °E and 45.05 °N, 11.88 °E) from 1986 to 2014 (29 consecutive years), resulting in a dataset of 1296 records. Each record includes all the information in Table 1. The fields surveyed represent a balanced sample of agronomic conditions in northeast Italy. All the entomological (collection of larvae, species determination) and agronomic (crop stand and damage, cultivation practises, yield) information was directly collected with at least six inspections per field each year. Just 6 % of the rotation and landscape data was from farmer interviews (i.e., previous year data regarding fields reported for the first time and new to the authors). Data for the other parameters were collected from official regional databanks (see Soil properties). The majority of the fields surveyed were untreated (no soil insecticide or insecticide-coated seeds), except for those seeded alternately, with untreated and treated maize in strips/plots (particularly where higher wireworm densities were recorded with bait trapping method [Furlan 2014]), and those inspected after severe damage was reported and found to be treated with soil insecticides. Land treated with soil insecticides (including insecticide-coated seeds) accounted for 3.96 % of the total land surveyed. Every year, any field suffering from newly reported wireworm damage was added to a database of farms being continuously surveyed (about 100 ha a year with few exceptions) in order to increase the records for severe damage. These additional fields accounted for 8.15 % of the entire dataset. A total surface area of 4646 ha was studied over 29 years; the mean was 160 ha per year with a SD of 121.3 ha. A minimum of 15 ha was studied in 1991 and a maximum of 489 ha in 2014. Therefore, the survey comprised a random sample of maize fields in the investigated area with a moderately higher incidence of cases of considerable wireworm damage.

**Table 1:** List of the variables included in the database

Risk factors	Variable	Type	Classification	Maize cultivated land (ha)	(%)
Site identification	Year	Qualitative	1986–2014		
	Farm	Qualitative			
	Municipality	Qualitative			
	Province	Qualitative	BL, PD, RO, TV, VE, VI, GO, PN, UD		
	Region	Qualitative	Veneto, Friuli Venezia Giulia	4509.69, 136.7	
	Plot	Qualitative			
	Field	Qualitative			
	GPS coordinate *y*				
	GPS coordinate *x*				
	Land cultivated with maize for assessment (ha)	Quantitative		4646.39	100
Crop damage	Damage index: total plant damaged by wireworms (seed + emerged) (%)	Quantitative	0–5.00	4219.49	91.03
			5.01–15.00	215.03	4.63
			15.01–50.00	164.83	3.55
			50.01–80.00	32.84	0.71
			≥80.01	4.2	0.09
	Yield assessment	Qualitative	Yes	42.58	0.92
			No	293.78	6.32
			NA	4310.03	92.75
	Yield reduction (t/ha)	Quantitative	No	293.77	6.32
			≤2.00	15.32	0.33
			2.01–3.00	25.69	0.55
			≥3.01	1.73	0.04
			NA	4309.88	92.76
Soil properties	Organic matter (%)	Quantitative	0.00–2.00	725.72	15.62
			2.01–5.00	3735.77	80.40
			≥5.01	184.90	3.98
	Texture	Qualitative	C (clay)	221.83	4.77
			L (loam)	769.23	16.56
			CL (clay loam)	7.10	0.15
			Csilt (clay silt)	3584.28	77.14
			LS (loam sand)	61.03	1.31
			S (sand)	2.92	0.06
	Skeleton (%)	Quantitative	0.00	4498.73	98.82
			0.01–15.00	24.11	0.52
			15.01–35.00	76.55	1.65
			≥35.01	47.00	1.01
	Drainage	Qualitative	WD (well drained)	4563.40	98.21
			PD (poorly drained)	82.99	1.79
Agronomic practises	Rotation	Qualitative	A: continuous maize cultivation (at least four subsequent years before the year of the field assessment).	268.96	5.79
			B: different crops in a flexible order alternated with maize.	3762.67	80.98
			C: including double crops (e.g., soybean/sorghum after barley or canola) and/or meadow	614.75	13.23
	Main crop: 1 year before	Qualitative	Sugar beet	349.06	7.51
			Canola	61.91	1.33
			Winter wheat	409.09	8.80
			Sunflower	15.17	0.33
			Ryegrass	17.00	0.37
			Maize	1580.63	34.02
			Alfalfa	53.27	1.15
			Vegetables	0.23	0.00
			Barley	183.81	3.96
			Meadow	19.81	0.43
			Set aside	28.91	0.62
			Soybean	1908.38	41.07
			Sorghum	6.52	0.14
			Triticale	12.60	0.27
	Second crop: 1 year before	Qualitative	No	4421.80	95.17
			Yes	224.58	4.83
	Second crop: 2 years before	Qualitative	No	4608.16	99.18
			Yes	38.23	0.82
	Second crop: 3 years before	Qualitative	No	4573.85	98.44
			Yes	72.54	1.56
	Second crop: 4 years before	Qualitative	No	4615.06	99.33
			Yes	31.33	0.67
	Meadow and/or double crop within the two previous years	Qualitative	No	4220.68	90.84
			Yes	425.71	9.16
	Cover crops	Qualitative	No	4523.27	97.35
			Yes	123.12	2.65
	Sowing date	Qualitative	Ordinary	4614.96	99.32
			Late	31.43	0.68
Landscape	Landscape within 200 m around the considered field	Qualitative	LA: continuous maize cultivation (at least four subsequent years before the year of the field assessment)	187.86	85.98
			LB: different crops in a flexible order alternated with maize: soybean, winter	3995.01	4.04
			LC: including double crops (e.g., soybean/sorghum after barley or canola) and/or meadow or uncultivated grasses	463.52	0.99
Climate	Rainfall class^a^	Quantitative	>1 (Class =1)	1707.81	36.76
			≤1 (Class =0)	2938.57	63.24
	Mean spring temperature (°C)	Quantitative	>16 (Class =1)	2596.55	55.88
			≤16 (Class =0)	2049.83	44.12
	Mean spring temperature (2 years before) (°C)	Quantitative	>16 (Class =1)	2518.32	54.20
			≤16 (Class =0)	2128.06	45.80
	Mean annual temperature (°C)	Quantitative	>14 (Class =1)	1916.43	41.25
			≤14 (Class =0)	2729.95	58.75
Entomology	Main wireworm species found on damaged plants	Qualitative	*A. brevis*	396.33	8.53
			*A. litigiosus*	6.50	0.14
			*A. sordidus*	1104.36	23.77
			*A. ustulatus*	3139.20	67.56

*NA* not assessed

^a^Rainfall class =1 if spring rainfall of the station in planting season is > mean spring rainfall of the station recorded in 30 years (ratio >1); 0 if spring rainfall of the station in planting season is ≤ mean spring rainfall of the station recorded in 30 years (ratio ≤1)

### Damage assessment

When random untreated maize strips/plots (3 or 4.5 m wide) had been sown alternately with treated strips/plots, the most effective insecticides available were used (Table 2).

**Table 2:** List of soil insecticides used on the fields during the years of monitoring

Year	Product	AI	Dose	Type
1986–1994	Dyfonate^®^	Fonofos 4.75 %	10 kg/ha	granules applied in-furrow
	Dotan^®^	Chlormephos 4.95 %	7 kg/ha	granules applied in-furrow
1995–2005	Regent TS^®^	Fipronil	0.6 mg/seed	coating
	Gaucho^®^	Imidacloprid	1.2 mg/seed	coating
	Regent 2G^®^	Fipronil 2 %	5 kg/ha	granules applied in-furrow
2006–2010	Poncho^®^	Clothianidin	0.5 mg/seed	coating
2011–2013	Poncho^®^	Clothianidin	0.5 mg/seed	coating
	Santana^®^	Clothiadinin 0.7 %	11 kg/ha	granules applied in-furrow
	Gaucho^®^	Imidacloprid	1.2 mg/seed	coating
2014	Sonido^®^	Thiacloprid	0.5 mg/seed	coating

One litre of the fungicide Celest^®^ XL (metalaxil-m + fludioxonil) per ton of seed was mainly used to treat all the maize seeds planted. At the 2–3 and 6–8 leaf stages, two sub-plots of 20 m × 4 rows of maize per portion of untreated field (0.1–0.2 ha) or untreated strip (3–6 m × 100–300 m) were chosen at random and the plants observed. During plot trials, in the two central rows of each untreated plot, all plants were counted and divided into “healthy” and “damaged”; the plots covered an area of 15–18 m × 1.5 m. The location and the number of sub-plots were the same in both the untreated/treated strips and completely untreated fields. In order to assess wireworm damage on emerged plants, typical symptoms (e.g., wilting of central leaves, broken central leaf due to holes in the collar, wilting of whole small plants) were assessed and the soil around the unhealthy plants was dug up to a depth of 5–6 cm; any larvae found near the collar were collected and identified. Wherever maize plants were missing from the rows, the soil was dug up in order to assess possible wireworm damage to seeds and/or emerging seedlings. Total plant damage was calculated as the sum of damaged emerged plants and seeds. In order to establish the effect of wireworm damage on yield, the same observations were made on the treated strips/plots, when used. Finally, the strips and the plots were harvested and the maize grain weighed. Maize grain samples were collected and their humidity measured with a Pfeuffer-Granomat (the same machine was used for all samples each year). The four fields in which maize stands were irregular and damaged due to factors other than wireworm activity (e.g., bird damage, low emergence due to low soil moisture, flooding) were not considered. In order to isolate the “wireworm damage effect”, analysis excluded the five fields under considerable pressure from factors other than wireworms (e.g., other parasites such as viruses or rootworms, *Diabrotica virgifera virgifera* LeConte). Fields in which the general conditions were good, but the soil insecticide had not worked properly and the stand of treated maize plots was not optimal were not used to evaluate the effect on yield (two cases only). Only damage assessments from untreated strips were registered in the database (the mean of the sub-plot assessments for each considered field). When farmers reported wireworm outbreaks, we included any fields that could be inspected within the sixth leaf stage whenever damage could be reliably assessed and the species identified (95 % of reported cases were included). Larvae collected from damaged plants were used to attribute the damaging species to each record; fields were included in the database when a minimum of three larvae was found. In most cases, dozens of larvae were collected and identified; if more than one species was found, the one with the most individuals was considered the species responsible for the damage. Larvae were identified with specific keys (Furlan 1999; Rudolph 1974).

### Soil properties

The following characteristics were assessed for each field: organic matter content (OM), pH, and texture, according to the dataset of the Veneto Region’s Environmental Protection Agency (ARPAV). The local soil map geo-database contains all data from samples collected during soil field descriptions to map soils; these data were analysed by ARPAV. Soil from each surveyed field was classified based on the organic matter content, pH and texture, according to the soil characteristics of its soil map unit (SMU).

#### Organic matter

Organic carbon (OC) was established by wet oxidation followed by titration; OM was obtained by multiplying OC by 1.724 (Van Bemmelen factor).

#### pH

Analysis was conducted via potentiometric measurement of pH in aqueous dispersion 1:2.5 soil/water ratio.

#### Texture

Soils were classified with the USDA soil texture triangle based on analyses conducted with a sedimentation pipette (USDA 2014).

#### Drainage

Soil drainage was assessed by experts during field observations and profile descriptions; soil was assigned to drainage classes according to the USDA soil manual (USDA 2014). Fields were divided into two groups:

- Poorly drained (PD): most of the soil is wet at shallow depths periodically during the growing season or remains wet for long periods (mainly because of poor field layout); excess water after rain drains slowly, flooding generally occurs up to several times a year.
- Well drained (WD): good field layout; water is removed quickly from the soil and flooding is rare.

### Agronomic practises

The common agronomic practises applied to all the fields studied were the following: fertilization with 240–300 kg N per ha, 70,000 to 80,000 seeds/ha, inter-row width 75 cm, pre-emergence plus post-emergence herbicide treatments causing very low weed densities. The following commercial hybrids were used: ANITA, COSTANZA, ALICIA, SENEGAL (1993–2001); TEVERE (2002–2004); DKC6530 (2005–2006); DKC6530, MITIC, KERMESS, KLAXON (2007–2008); DKC6666, NK FAMOSO, PR31A34, PR32G44 (2009–2010); DKC6677, PR32G44, NK FAMOSO (2011); KORIMBOS, KALIPSO, P1547 (2012–2014).

#### Rotation

Rotation was classified into three groups:

- Rotation type A (Rot A): continuous maize cultivation (at least 4 years prior to the year of field assessment).
- Rotation type B (Rot B): several different crops in a flexible order alternated with maize. Soybean, winter cereals, sorghum and canola were the main ones; sunflowers, horticultural crops and potatoes were cultivated less, but featured in some records. Meadows and double crops were excluded.
- Rotation type C (Rot C): including double crops (e.g., soybean/sorghum after barley or canola) and/or meadows (e.g., alfalfa, *Festuca* spp., etc.); only meadows ploughed in the 3 months before the sowing were considered because ploughing presumably reduces attacks on seedlings/plants only when made not long before sowing.

#### Cover crops

In some cases, the main crops were alternated with cover crops: sudangrass hybrid (*Sorghum bicolor* (L.) Moench. × *Sorghum sudanense* [Piper] Stapf) during spring-summer, and a mixture of vetch (*Vicia sativa* L.) and barley (*Hordeum vulgare* L.) in fall-winter.

#### Sowing date

Sowing dates were divided into two groups:

- Ordinary sowing date: between late March and 30 April.
- Late sowing date: May or later.

#### Tillage

The vast majority of the fields considered for the database were tilled conventionally: ploughing, cultivator passages, harrowing and hoeing. Only 41 fields were under minimum tillage (consisting in a cultivator passage, harrowing and hoeing), while no sod-seeding field was included in the database.

### Landscape

The surrounding landscape was analysed. When more than 30 % of the land within 200 m was meadows (in rotation or permanent) or uncultivated land with grass and/or rotation causing continuous plant coverage (e.g., double crops as described above for Rot C), the record was classified as Landscape C (LC), whatever the rest of the land was used for. When the prevalent surrounding condition was as described for Rot A (i.e., Rotation), the record classification was Landscape A (LA), and when as described for Rot B, the record classification was Landscape B (LB).

### Climate

The effect of rainfall and temperatures on wireworm damage was assessed by using 30 years of climatic series from the closest ARPAV meteorological station in order to give a complete description of each dataset (Table 1). All surveyed fields were located between 1 and 14 km from their nearest meteorological station, but most of them were less than 5 km away.

Rainfall class 1/0 was established by comparing the spring rainfall of a given station in planting season with the mean spring rainfall of the station recorded in 30 years, corresponding to:

- class 1 when the former was higher than the latter (rate >1);
- class 0 when it was lower or equal to (rate ≤1).

Temperatures were also dichotomized as follows:

- spring mean temperature (30 years) of the station closest to field locality versus spring mean temperature of all stations (16 °C);
- spring mean temperature (2 years before planting) of the station closest to field locality versus mean spring temperature of all stations (16 °C); and
- annual mean temperature (30 years) of the station versus annual mean temperature of all stations (14 °C).

As rainfall, temperature parameters were classified by assigning 1/0 for temperatures above/below the mean value.

### Statistical methods

Analysis was performed by SAS 9.3 (SAS Institute Inc., Cary, NC). All statistical models included observed land surface area as a weight variable.

A logistic regression on a dichotomous variable (reduction in yield [Yes =1/No =0]) was performed on 213 records (333 ha) to estimate the probability of production loss based on the percentage of damaged plants. The Youden criterion was used to set the optimum cut-off point for the level of plant damage above which yield reduction was deemed to be significant (Lambert and Lipkovich 2008). Yield reduction (t/ha) was analysed using a one-way ANOVA linear model which used the percentage of damaged plants as a fixed effect (<15 %; 15–20 %; 20–30 %; 30–40 %; >40 %).

According to the threshold calculated in the previous step, all data underwent risk analysis on a dummy variable, i.e., the percentage of damaged plants (1= percentage of damaged plants greater than threshold; 0= percentage of damaged plants less than or equal to threshold); we used a generalized linear model with binomial distribution and a log-link function. The log-binomial regression was used to analyse a dichotomous response variable and to model outcome probability (e.g., probability of disease or damage), given the exposure to sources of risk and confounders. The model equation is defined as follows:

1 $$ \ln \left(\pi (x)\right)={\beta}_0+{\beta}_1x $$

Where *π*(*x*) is the probability of the event (damage); *β*_0_ is the intercept of the model; *β*_1_ is the regression coefficient; and *x* is the predictor (risk factor).

The model assumes that the error terms have binomial distribution.

Model parameterization dictates that exp(*β*_1_) is the relative risk for a one-unit increase of the independent predictive *x* variable (risk factor) when it is continuous, or for the presence of a higher risk level when *x* is a categorical factor (Spiegelman and Hertzmark 2005). Damage probability for every risk factor was estimated as a percentage of surface exposed to risk where damage was recorded (prevalence of damage). The ratio between the percentage of surface exposed to risk and surface not exposed to risk, when damage was recorded in both cases, gave the risk ratio (RR). For the log-binomial regression model, RR calculated as a ratio of prevalences is equal to RR calculated as exp(*β*_1_). When this rate was significantly greater than 1 for any level of risk factor, it meant that this level of risk factor increased the probability of damage significantly. All risk factors were dichotomized, with 1 representing the expected higher risk level. A univariate approach was adopted to select the potential predictor risk factors based on a P level (*P* < 0.05).

Homoscedasticity and independence of the residuals were graphically evaluated by plotting the standardized residuals versus the predicted values. No significant deviation from the hypothesis was observed.

Predictive variables were checked for collinearity and a kappa index for pairs of dichotomous variables was used as an indicator of association between risk factors. Lastly, multivariate risk analysis was conducted by using a multifactorial model that included all the significant variables from the previous univariate step and which had also been registered for all records (*n* = 1259). The multifactorial model was a log-binomial regression model and the equation was similar to (1) with additional terms which accounted for the contribution of every additional risk factor. The risk ratios were adjusted for the model’s continuous and categorical covariates and estimated by Poisson regression with robust error variance (Zou 2004).

## Results and discussion

### Wireworm damage on maize yield—economic damage thresholds

The main species found were *A. brevis*, *A. sordidus* and *A. ustulatus*. All of these species are widespread in central and southern Europe (Furlan 1996, 2004; Furlan and Tóth 2007), including areas with significantly different conditions from Veneto, e.g., in Austria, where *A. brevis* was found in zones with acid pH (Staudacher et al. 2013). *A. brevis* and/or *A. sordidus* were responsible for all types of damage (seed erosion and leaf wilting) even on developed maize plants (8–10 leaves stage); most of the damaged plants had one or more wilted central leaves due to larval feeding on the collar, which sometimes killed them, while *A. ustulatus* affected plant stand only by damaging seeds as described by Furlan (2014). Plant damage was partially (sometimes completely) compensated by the growth of sprouts from the plant collar and by a general increase in the mean cob weight in the field area where the stand had been significantly reduced by wireworm damage.

**Fig. 1:**
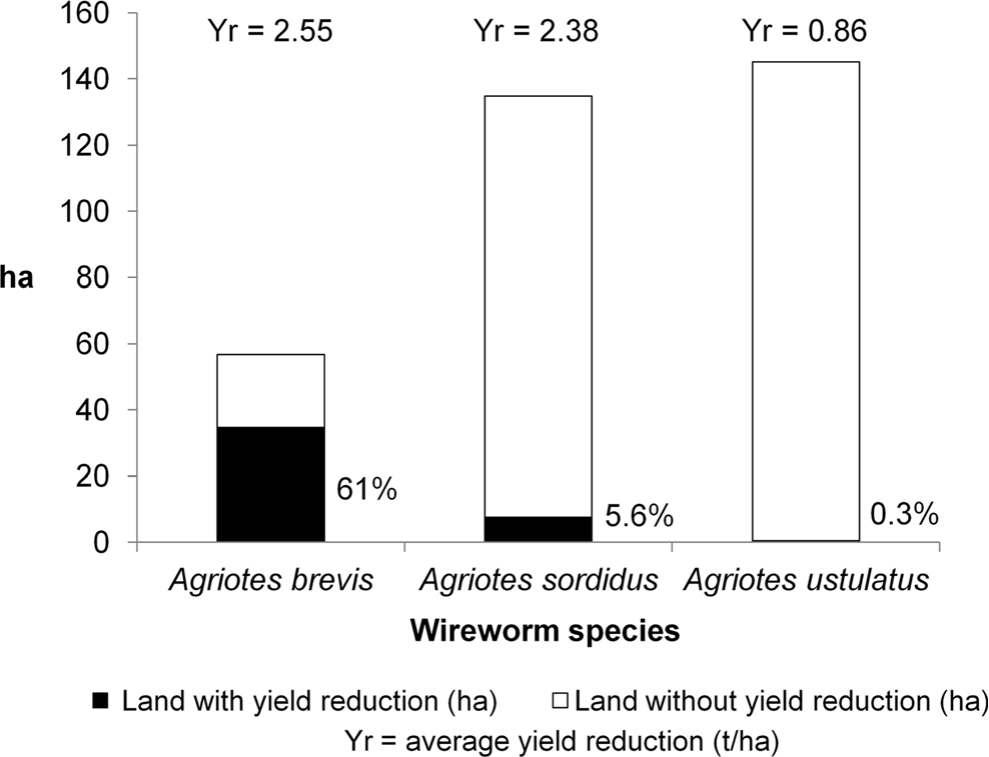
Potential harmfulness of the *Agriotes* species. Incidence of yield reduction for fields with over 15 % plant damage

The Youden criterion of logistic model establishes 20 % of damaged plants as optimal cut-off to discriminate a significant increase of probability in yield reduction. However, we prudently observed that over the 29-year period surveyed, no yield reduction was caused when wireworm plant damage was below 15 % of the stand (risk of yield reduction approximately zero), regardless of the hybrid and agronomic/climatic conditions. More than 91 % of the land with below 15 % plant damage had negligible damage (<5 %), and at least 25 % of the damaged plants in all fields fully recovered, as after 30 days the damaged plants could not be distinguished from the undamaged ones. Less than 20 % of the attacked plants were dead or stunted, and the remainder produced ears that were either smaller or normal but had slightly higher moisture content. Furthermore, it was rare to find more than two consecutive damaged plants in a row in these fields. This resulted in a final homogeneous stand with more than 90 % of the seeds sown developing into a productive plant. All of these fields (less than 15 % damage) produced the expected stand which varied per hybrid from 6 to 7.5 plants/m^2^. In conclusion, wireworm activity caused no negative impact on yield. The probability of yield reduction rose when the percentage of damaged plants increased and became considerable when plant damage was over 25 % (Table 3). More prudently, 15 % of damaged plants was considered the threshold for discriminating cases with or without risk of yield reduction due to wireworm attacks on maize. Potential damage varied by species, with *A. brevis* being mainly the most harmful (Fig. 1).

**Table 3:** Effect of wireworm damage on maize yield as a percentage of the number of plants attacked (any symptom) on maize yield. Dataset covers 29 years

Plant damage (%)	No. of cases observed	Land (ha)	No. of yield reduction cases	Land with yield reduction (ha)	Mean yield reduction (t/ha) (LS-means ± se)^a^	Probability of yield reduction (range) %	Probability of yield reduction (mean) %	Risk ratio	95 % confidence interval	P chi-square test
**<15**	151	260.85	0	0	–	0–1	0.4	ne	ne	ne
**15–20**	11	20.32	2	1.2	1.28 ± 0.63 ***b***	2–16	7	0.43	0.07–2.52	0.3195
**20–30**	19	11.67	11	4.88	1.86 ± 0.31 ***b***	17–62	37	3.46	1.65–7.24	0.0031
**30–40**	9	5.97	6	4	2.43 ± 0.34 ***a***	62–94	81	5.51	2.92–10.39	<0.001
**>40**	23	34.17	22	33.67	2.72 ± 0.12 ***a***	>94	98	29.21	15.90–93.66	<0.001
**Tot**	*213*	*332.98*	*41*	*43.75*						

*ne* not estimable

^a^LS-means ± se = least square means ± standard error; means with different bold letters are significantly different for *P* < 0.05

### General effect of damage risk factors including all species

#### Soil organic matter

Organic matter content was the strongest risk factor (RR =31.94, *P* < 0.0001; Table 4). When OM content was over 5 %, the risk of damage considerably and significantly increased. This cannot be attributed to the potential of soil organic matter to feed wireworms (Furlan 1998, 2004; Traugott et al. 2008), as previously believed (Salt and Hollick 1946), but it is probably due to the general condition of soils that are rich in organic matter (e.g., more weeds and prolonged high moisture content), which may result in a higher survival rate for small larvae. Kozina et al. (2015) reported that humus content (%) was the best predictor of high *Agriotes lineatus* L. populations. When humus content was >4.65 %, a very high density was predicted, but the authors refer to click beetles captured by pheromone traps, and do not supply plant damage data. Therefore, the information has no practical implications on risk assessment. Saussure et al. (2015), however, did not find that higher soil OM content led to increased damage risk, but they did not provide absolute data analyses. Consequently, it is not possible to ascertain whether OM values in France ranged below or above the threshold found in this study. Furthermore, they did not separate data for the various wireworm species involved. Poor drainage resulted in significantly higher damage risk as well (RR =3.75, *P* < 0.0001; Table 4). *A. brevis* was favoured by high OM content and poor drainage (this species was the prevalent wireworm in over 90 % of the seriously damaged fields in these conditions).

**Table 4:** Effect of different potential risk factors on crop damage (discriminating value 15 % of damaged plants), considering three main wireworm species

Risk factors	Characteristics	Comparisons	Records (*n*)	% of land with plant damage >15 % (prevalence of damage)	RR	se	Wald chi-square	*P*
Soil properties	Organic matter	>5 % vs ≤5 %	1296	66.39	31.94	3.67	909.90	<0.0001
	Texture	C vs others	1259	7.36	3.83	1.32	15.19	<0.0001
		L vs others		3.46	1.93	0.44	8.32	0.0039
		CL vs others		37.04	18.28	10.43	25.93	<0.0001
		Csilt vs others		1.56	0.39	0.08	20.53	<0.0001
		LS vs others		0.83	0.40	0.60	0.38	0.5379
		S vs others		9.25	4.48	8.23	0.67	0.4140
	Skeleton	>0 vs 0	1259	9.07	4.91	1.43	29.71	<0.0001
	Drainage	PD vs WD	1296	16.43	3.75	0.96	26.42	<0.0001
Agronomic practises	Rotation	A vs others	1259	1.31	0.62	0.34	0.77	0.3813
		B vs others		1.08	0.17	0.04	73.19	<0.0001
		C vs others		8.57	7.83	1.59	102.06	<0.0001
	Main crop: 1 year before	Winter wheat vs others	1176	5.23	4.75	1.28	33.52	<0.0001
		Maize vs others		0.97	0.57	0.17	3.55	0.0596
		Alfalfa vs others		12.64	9.48	3.89	30.08	<0.0001
		Barley vs others		13.96	10.63	4.05	38.52	<0.0001
		Meadow vs others		9.32	6.56	4.73	6.81	0.0091
		Soybean vs others		0.53	0.24	0.08	17.12	<0.0001
		Set aside vs others		8.62	6.27	3.07	14.03	0.0002
		Canola vs others		1.73	1.19	1.67	0.02	0.9018
	Second crop: 1 year before	Yes vs No	1259	14.80	10.17	2.13	122.64	<0.0001
	Second crop: 2 years before	Yes vs No	1259	24.30	12.74	4.20	59.69	<0.0001
	Second crop: 3 years before	Yes vs No	1259	11.17	5.82	2.03	25.59	<0.0001
	Second crop: 4 years before	Yes vs No	1259	9.57	4.75	2.66	7.78	0.0053
	Meadow and/or double crop within the 2 previous years	Yes vs No	1259	11.79	10.44	2.10	136.14	<0.0001
	Cover crops	Yes vs No	1259	6.76	3.49	1.24	12.31	0.0005
	Sowing date	Late vs ordinary	1259	10.50	5.23	2.78	9.69	0.0018
Landscape	Landscape within 200 m around the considered field	C-LC^a^ vs others	1259	13.52	5.27	1.23	50.53	<0.0001
		Others-LC^b^ vs others		6.70	2.07	0.84	3.19	0.0450
		C-L^c^ others vs others		2.64	0.60	0.24	1.66	0.1979
		Others-L^d^ others vs others		0.95	0.15	0.04	57.42	<0.0001
Climate	Rainfall class^e^	>1 vs ≤1	1259	2.42	1.31	0.27	1.68	0.1943
	Mean spring temperature (°C)	>16 vs ≤16	1259	2.47	1.58	0.34	4.45	0.0350
	Mean spring temperature (2 years before) (°C)	>16 vs ≤16	1259	2.35	1.3728	0.2922	2.22	0.1367
	Mean annual temperature (°C)	>14 vs ≤14	1259	2.23	1.1364	0.2358	0.38	0.5376
Entomology	Species damaging maize	*A. brevis* vs others	1259	14.36	10.46	2.14	131.68	<0.0001
		*A. sordidus* vs others		4.34	3.28	0.67	33.49	<0.0001
		*A. ustulatus* vs others		0.33	0.05	0.02	79.14	<0.0001

^a^C-LC vs others = Rotation C in the field and LC landscape vs any other combination

^b^Others-LC vs others = Rotation A or B in the field and LC landscape vs any other combination

^c^C-L others vs others = Rotation C in the field and LA or LB landscape vs others vs any other combination

^d^Others-L others vs others = Rotation A or B in the field and LA and LB landscape vs any other combination

^e^Rainfall class = >1 if spring rainfall of the station in planting season is > mean spring rainfall of the station recorded in 30 years; ≤1 if spring rainfall of the station in planting season is ≤ mean spring rainfall of the station recorded in 30 years

#### Soil pH

Soil pH was alkaline for most fields and ranged from 7.9 to 8.4, as all fields were in an area where soils have high percentages of calcium and magnesium carbonates (20–50 %). Only some fields were in depressed areas with peaty soils; in these few cases, soils were acid and pH well below 7.

#### Soil texture

Regarding other soil properties (Table 4), clay (C), loam (L) and clay loam (CL) soils increased the damage risk, as did the presence of skeleton in soils (RR =4.91, *P* < 0.0001). Although larvae can move more easily and faster in sandy soils, with high damage potential when they occur in considerable densities, it is likely that higher clay content soils may retain higher humidity for longer, thus causing lower egg and larval mortality in the first and most sensitive phases of the insects’ life cycle (Furlan 1998, 2004). This difference may become negligible when sandy soils are irrigated.

#### Rotation

Rotation including meadows and second crops significantly increased damage risk (Rot C vs others: RR =7.83, *P* < 0.0001; Table 4). This may be because the soil is continuously covered by growing plants, resulting in more roots for small larvae to feed on, thus less movement and risk of starvation (Furlan 1998, 2004; Traugott et al. 2008).

Any type of meadow and combination of second crops sown within 2 years prior to maize cultivation strongly and significantly (RR =10.44, *P* < 0.0001) increased damage risk. As per a number of previous observations (Furlan 1998, 2004; Szarukán 1977), these factors resulted in lower mortality and higher wireworm populations for the next maize crops. Although meadows and double crops appear to be key factors, differences were found among other crops. Using soybean as a previous crop resulted in a much lower risk than any other previous crop, while winter cereals such as winter wheat (RR =4.75, *P* < 0.0001) and barley (RR =10.63, *P* < 0.0001) were more likely to favour plant damage over 15 % than maize or the other main crops studied. Using cover crops in rotation increased damage risk (roughly triple), but it appears to be a weaker agronomic factor than others.

#### Sowing date

Late sowing significantly increased damage risk (RR =5.23, *P* = 0.0018) compared with the ordinary sowing date (Table 4). This result was seemingly controversial, as higher temperatures lead to quicker growing phases, which should allow seedlings/small plants to resist wireworm attacks more effectively. It may be explained by biological factors, in that late sowing implies higher temperatures and a shorter time span for moulting larvae. In these conditions, a larger part of the population may moult and enter the feeding phase (Furlan 1998, 2004), while small plants are still susceptible. Therefore, more larvae would attack the plants.

#### Tillage

The 41 fields cultivated with minimum tillage did not show any plant damage increase compared with conventional tillage practises. Indeed, none of these fields experienced plant damage over 10 %. In six of these fields, the yield of untreated/treated strips was assessed; no differences were found, as expected (see Soil properties). Due to lack of fields with plant damage over 15 %, it was impossible to calculate RR in this case. Saussure et al. (2015) found that the number of tillage operations, especially in spring, raised damage probability, but did not make clear whether these operations were part of conventional procedures, including ploughing. In our case, the number of tillage operations (cultivation, harrowing, hoeing) did not vary significantly between farms in the area studied, which meant that this factor could not be used to evaluate damage risk.

#### Landscape

The presence of an LC landscape (meadows, second crops, uncultivated grass) around the field considered significantly influenced wireworm plant damage risk for fields with a low-risk rotation (Rot A and Rot B); when Rot C and LC were combined, the risk of damage was much higher (Table 4). These results confirm observations reported by other authors (Blackshaw and Hicks 2013; Benefer et al. 2012; Hermann et al. 2013; Saussure et al. 2015).

#### Climate

Rainfall and temperatures did not influence risk particularly, although temperatures above 16 °C in the same spring of maize cultivation increased damage probability (RR =1.58, *P* = 0.035; Table 4). Higher temperature may allow larvae to moult more quickly and then increase the number of larvae in a potentially harmful feeding phase (Furlan 1998, 2004). The low impact of climatic factors on risk of wireworm damage may be explained by the limited variations in climate between the sites monitored, all of which were located in a fairly homogeneous area. Stronger impact on population levels was described by other authors (Kozina et al. 2015; Staudacher et al. 2013), who compared sites with significant climatic differences.

### Effect of species on damage risk

#### *Agriotes brevis*

We found that the percentage of total damage variability for this species (Table 5) was mainly explained by rotation, i.e., the presence of double crops or meadows within 2 years of maize being sown.

**Table 5:** Effect of different potential risk factors on crop damage (discriminating value 15 % of damaged plants), considering the species *Agriotes brevis*

Risk factors	Characteristics	Comparisons	Records (*n*)	% of land with plant damage >15 % (prevalence of damage)	RR	se	Wald chi-square	*P*
Soil properties	Texture	C vs others	116	18.49	1.32	0.71	0.26	0.6087
		L vs others		12.19	0.75	0.24	0.8	0.371
		CL vs others		100.00	Not estimable			
		Csilt vs others		15.51	1.14	0.36	0.18	0.6701
		LS vs others		3.06	0.21	0.37	0.77	0.3817
	Skeleton	>0 vs 0	116	12.80	0.83	0.27	0.32	0.5712
Agronomic practises	Rotation	A vs others	116	1.85	0.09	0.07	9.4	0.002
		B vs others		14.40	1.00	0.45	<1	0.994
		C vs others		23.45	4.36	1.81	12.5	<0.0001
	Main crop: 1 year before	Winter wheat vs others	83	22.08	2.54	1.22	3.78	0.0519
		Maize vs others		3.24	0.12	0.07	14.39	0.0001
		Alfalfa vs others		53.85	5.88	2.82	13.67	0.0002
		Meadow vs others		17.54	1.69	1.59	0.31	0.5753
		Soybean vs others		36.94	4.34	2.03	9.89	0.0017
	Second crop: 1 year before	Yes vs No	116	21.67	2.04	0.64	5.2	0.0226
	Second crop: 2 years before	Yes vs No	116	42.64	3.14	1.51	5.67	0.0173
	Second crop: 3 years before	Yes vs No	116	10.48	0.69	0.35	0.53	0.4677
	Meadow and/or double crop within the two previous years	Others-others	116	23.92	4.10	1.62	12.79	0.0003
Landscape	Landscape within 200 m around the considered field	C-LC^a^ vs others	116	25.98	3.72	1.85	6.99	0.0082
		Others-LC^b^ vs others		44.48	7.62	4.34	12.75	0.0004
		C-L^c^ others vs others		5.24	0.44	0.51	0.51	0.4766
		Others-L^d^ others vs others		1.46	0.08	0.07	8.27	0.004
Climate	Rainfall class^e^	>1 vs ≤1	116	6.93	0.25	0.08	16.87	<0.0001
	Mean spring temperature (°C)	>16 vs ≤16	116	13.83	0.87	0.30	0.17	0.6843
	Mean spring temperature (2 years before) (°C)	>16 vs ≤16	116	20.08	2.2397	0.7646	5.58	0.0182
	Mean annual temperature (°C)	>14 vs ≤14	116	11.08	0.6787	0.2387	1.21	0.2704

^a^C-LC vs others = Rotation C in the field and LC landscape vs any other combination

^b^Others-LC vs others = Rotation A or B in the field and LC landscape vs any other combination

^c^C-L others vs others = Rotation C in the field and LA or LB landscape vs others vs any other combination

^d^Others-L others vs others = Rotation A or B in the field and LA and LB landscape vs any other combination

^e^Rainfall class ≥1 if spring rainfall of the station in planting season is > mean spring rainfall of the station recorded in 30 years; ≤1 if spring rainfall of the station in planting season is ≤ mean spring rainfall of the station recorded in 30 years

Rotation C increased damage risk over four times more than other rotation types, corroborating the estimated effect of using double crops or meadow within 2 years of maize being sown, which roughly quadrupled damage risk. The use of meadows (also as natural areas) and double crops around the studied fields significantly increased damage risk. Using alfalfa, soybean and winter wheat as previous year crops also increased risk, whereas maize reduced risk significantly (RR =0.12, *P* = 0.0001).

#### *Agriotes sordidus*

Rotation, particularly the use of double crops or meadows within 2 years, had less influence on *A. sordidus* (Table 6) than on *A. brevis*. Rotation C increased damage risk 2.60 times (*P* = 0.0007) more than other rotation types, similar to the estimated effect of planting double crops or meadow within 2 years of maize, which increased damage risk by 3.09 times (*P* < 0.0001). Soil texture also affected risk, with clay soils being prone to higher damage risk by *A. sordidus* (RR =3.59, *P* = 0.0024). Previous crops, e.g., alfalfa (RR =2.87, *P* = 0.067), canola and winter wheat, also raised damage risk, whereas soybean reduced it.

**Table 6:** Effect of different potential risk factors on crop damage (discriminating value 15 % of damaged plants), considering the species *Agriotes sordidus*

Risk factors	Characteristics	Comparisons	Records (*n*)	% of land with plant damage >15 % (prevalence of damage)	RR	se	Wald chi-square	*P*
Soil properties	Texture	C vs others	512	14.28	3.59	1.51	9.23	0.0024
		L vs others		3.83	0.84	0.28	0.27	0.6049
		CL vs others		0.00	Not estimable			
		Csilt vs others		4.25	0.95	0.28	0.03	0.8551
		LS vs others		0.34	0.08	0.19	1	0.3163
		S vs others		9.25	2.14	3.93	0.17	0.6795
Agronomic practises	Rotation	A vs others	512	2.04	0.45	0.34	1.14	0.286
		B vs others		3.27	0.49	0.14	6.32	0.012
		C vs others		8.17	2.60	0.73	11.54	0.0007
	Main crop: 1 year before	Winter wheat vs others	464	5.58	1.95	0.66	3.92	0.0479
		Maize vs others		3.86	1.12	0.41	0.09	0.7589
		Alfalfa vs others		9.65	2.87	1.65	3.35	0.0672
		Meadow vs others		5.97	1.69	1.85	0.23	0.6324
		Soybean vs others		0.84	0.17	0.10	8.5	0.0035
		Canola vs others		8.87	2.69	1.35	3.85	0.0497
	Second crop: 1 year before	Yes vs No	512	10.67	3.00	0.93	12.42	0.0004
	Second crop: 2 years before	Yes vs No	512	32.15	8.16	3.24	27.87	<0.0001
	Second crop: 3 years before	Yes vs No	512	26.36	6.55	2.94	17.53	<0.0001
	Second crop: 4 years before	Yes vs No	512	10.53	2.52	1.43	2.68	0.1019
	Meadow and/or double crop within the two previous years	Yes vs No	512	9.82	3.09	0.88	15.73	<0.0001
	Cover crops	Yes vs No	512	6.87	1.70	0.63	2.03	0.1539
	Sowing date	Late vs ordinary	512	10.73	2.57	1.45	2.8	0.0945
Landscape	Landscape within 200 m around the considered field	C- LC^a^ vs others	512	7.92	1.66	0.64	1.73	0.1884
		Others-LC^b^ vs others		3.93	0.65	0.52	0.28	0.5941
		C-L^c^ others vs others		8.92	1.95	0.95	1.86	0.1724
		Others-L^d^ others vs others		3.10	0.47	0.17	4.21	0.0403
Climate	Rainfall class^e^	>1 vs ≤1	512	4.95	1.26	0.36	0.69	0.4064
	Mean spring temperature (°C)	>16 vs ≤16	512	4.45	1.10	0.35	0.09	0.7681
	Mean spring temperature (2 years before) (°C)	>16 vs ≤16	512	4.38	1.0314	0.3217	0.01	0.9209
	Mean annual temperature (°C)	>14 vs ≤14	512	5.32	1.5745	0.456	2.46	0.117

^a^C-LC vs others = Rotation C in the field and LC landscape vs any other combination

^b^Others-LC vs others = Rotation A or B in the field and LC landscape vs any other combination

^c^C-L others vs others = Rotation C in the field and LA or LB landscape vs others vs any other combination

^d^Others-L others vs others = Rotation A or B in the field and LA and LB landscape vs any other combination

^e^Rainfall class ≥1 if spring rainfall of the station in planting season is > mean spring rainfall of the station recorded in 30 years; ≤1 if spring rainfall of the station in planting season is ≤ mean spring rainfall of the station recorded in 30 years

#### *Agriotes ustulatus*

Most of the factors did not show any significant effect on plant damage by *A. ustulatus* (data not shown). Some climatic parameters were the only factors to increase the damage risk ratio: the spring rainfall of the sowing year, which was higher than the mean spring rainfall over a 30-year period, increased the damage risk ratio by more than five times (RR =5.52, *P* = 0.0163); the spring temperature of the sowing year, which was lower than 16 °C, increased the damage risk ratio by almost six times (RR =5.91, *P* = 0.0504).

### Multifactorial model

No significant interactions between main factors were found, and all of them were included in the multivariable model. The estimated model was:

2 $$ \mathrm{In}\left(\uppi \left(\mathrm{y}\right)\right)=-6.05+2.65\kern0.1em OM+2.92\kern0.2em A.\kern0.2em brevis+1.73\kern0.2em A.\kern0.2em sordidus+1.19\kern0.5em \mathrm{sowing}\kern0.5em \mathrm{date}+0.96\kern0.5em \mathrm{cover}\kern0.5em \mathrm{crops}+1.64\kern0.5em \mathrm{rot}\kern0.5em C+1.65\kern0.5em LC $$

where *π*(*y*) is the probability of damage.

The predictors had a value of 1 in the presence of an exposure risk level (“>5 %” for OM; “Late” for sowing date; “Yes” for cover crops).

The generalized R square of the model was 35 %, and 83 % of cases were correctly classified, indicating that the parameterization was suitable.

Multivariate analysis of factors highlighted some change in the estimation of risk ratios for the independent contribution by variables included in the final model: RR of sowing date and cover crops decreased from 5.22 to 3.27 and from 3.49 to 2.60, respectively. Both factors were on the limit of statistical significance. The strongest factors (*P* < 0.001) were the following: *A. brevis* as the prevalent damaging species (RR from 14.36 to 18.51), OM (RR from 31.94 to 14.13), Rot C (RR from 8.57 to 5.16), *A. sordidus* as the prevalent damaging species (RR from 4.34 to 5.70) and landscape LC (others—LC) with RR increased from 2.06 to 5.18. All the other factors had no significant effects on wireworm damage risk.

## Conclusion

**Fig. 2:**
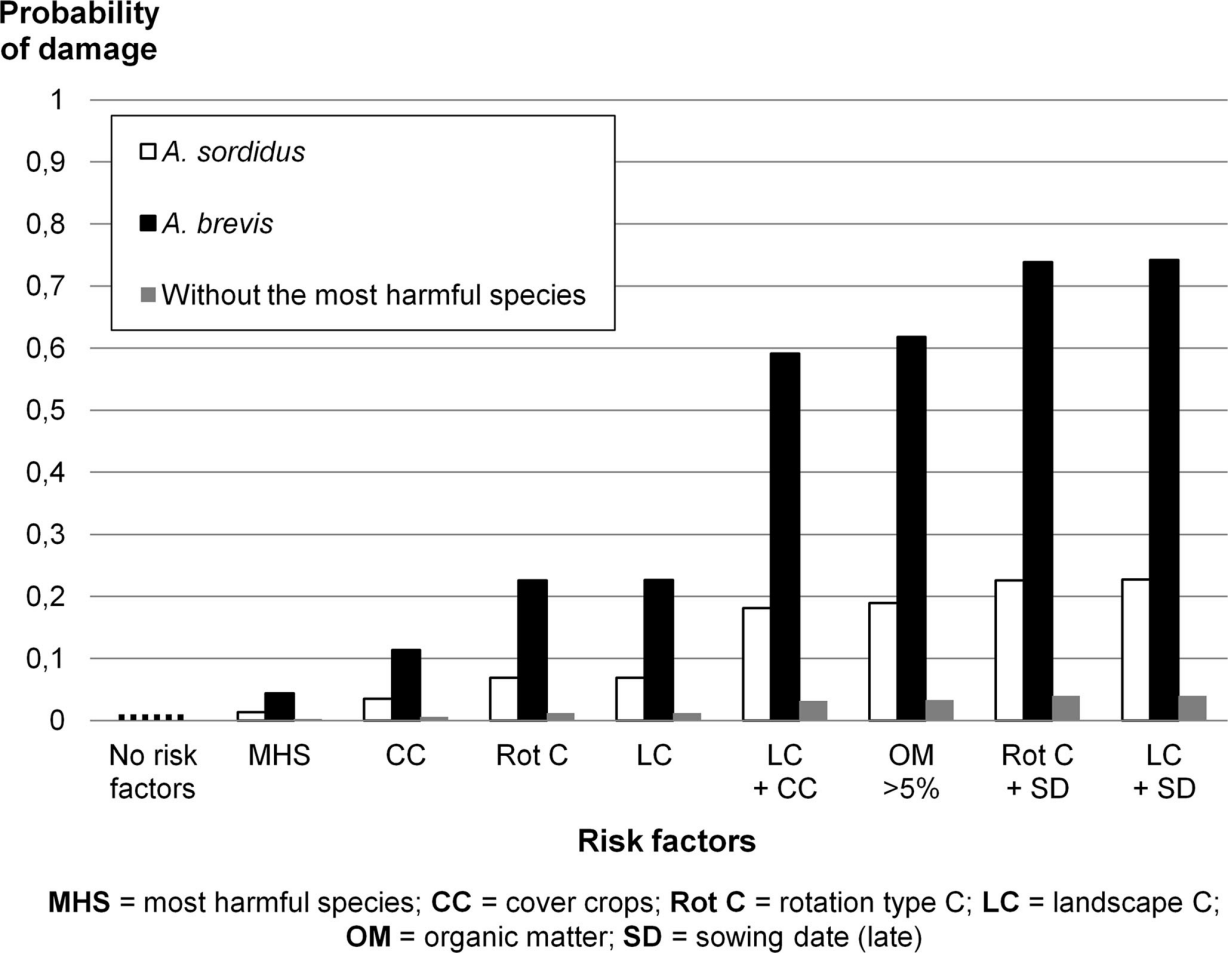
Estimated damage probability by wireworms based on multifactorial risk assessment analysis. Most harmful species (MHS) estimates damage probability in an area with the most harmful wireworms species and no other risk factors. All the other cases (CC, Rot C, etc.) represent damage probability in an area with one or none of the most harmful species plus a range of risk factors. No-risk factor gives the damage probability when neither the most harmful species nor other risk factors are present

This rich (>1200 records) set of long-term data has allowed us to prepare a practical list of the key risk factors affecting the probability of maize damage by wireworms. The prevalent *Agriotes* species play an important role, and *A. brevis* is potentially much more harmful than *A. sordidus* (Fig. 2).When an area contains none or a low level of the most harmful species (*A. brevis* and *A. sordidus*), damage probability is very low (0.2 % without any other risk factor; Fig. 2). Therefore, it is important to know an area’s key species and this may be achieved with pheromone traps at little cost (Burgio et al. 2012). Strong risk factors include organic matter content >5 %, rotations including meadows and alfalfa, double crops 1 or 2 years before maize is sown, and landscape around the maize fields including meadows and/or natural grass, alfalfa and double crops. Meadows had already been found to be a major risk factor, but wireworm presence needed more years to become conspicuous and actually harmful in England (Parker and Seeney 1997). This might be explained by the different species involved (*Agriotes lineatus* L., *Agriotes obscurus* L., *Agriotes sputator* L., Furlan et al. 2001; Furlan and Tóth, 2007; Salt and Hollick 1946) and by a different climate (e.g., lower temperatures). Weaker risk factors include poor field drainage, a late sowing date, a warm spring, cover crops and clay or clay-loam soils. The present information may be used to implement IPM and to tackle soil pests in many European regions and beyond. As a result, it may lead to a considerable reduction in the use of soil pesticides and to the immediate containment of the environmental impact of agriculture with no negative repercussions on farmers’ income. This can be achieved by implementing two steps:

1. “area-wide” risk assessment including click beetle population monitoring with pheromone traps;
2. “complementary field monitoring” where risk assessment has identified the presence of risk factors.

When a harmful population is found, whether damage really occurs or not may be forecast by wireworm-activity predicting models based on soil humidity and temperature (Jung et al. 2014; Milosavljević et al. 2016).

1. “Area-wide” risk assessment: the results of this work enable each cultivated region to be mapped, and high-risk areas to be pinpointed. Mapping the risk factors found in this survey and that of Saussure et al. (2015) outside Italy may enable us to prove that the cost-benefit analysis of past soil-insecticide use was extremely negative. The first layer of the map would include the main soil characteristics (organic matter content, pH, texture); the second would include the key agronomic characteristics (rotation, drainage); and the third, the available entomological information, such as click beetle population levels for the main *Agriotes* species, or wireworm presence/density assessed with bait traps over the years. This system could enable areas with different risk levels to be highlighted. Each wireworm-risk category (e.g., low, medium or high, based on the presence of one or more risk factors) will have its own IPM strategy, e.g., assessing wireworm density in high-risk areas, and opting not to treat or continue monitoring low-risk areas, possibly combined with mutual-fund insurance coverage (Ferrari et al. 2015). Figure 2 helps this approach as it shows how risk changes by combinations of risk factors. Once a country’s basic risk value is established (plant damage assessment of untreated no-risk fields), the risk indexes for individual areas can then be estimated, and bespoke IPM strategies suggested and implemented. The absence of risk factors greatly decreases the risk of economic damage and makes applying soil insecticides pointless (in most of the cases).
2. Complementary field monitoring: where risk factors are present, we suggest assessing actual wireworm populations using bait traps with the following procedure:
  i) in high-risk areas, assess current *Agriotes* populations with the procedure described in Furlan (2014) using bait traps that estimate the actual mean larval population in fields intended for maize sowing;
  ii) when the mean number of wireworms does not exceed the thresholds established, maize may be sown without any treatment;
  iii) when the mean number of wireworms exceeds at least one of the thresholds, farmers have the option of moving maize to a no-risk field, as well as of applying organic treatments (Furlan and Kreutzweiser 2015), or chemical treatments.

In this way, control strategies will be implemented only when and where economic thresholds for maize are exceeded.

### Benefits to other crops

The risk factors causing high wireworm populations in maize are the same as those in other crops. Therefore, they can be used to implement IPM in all arable crops with possible adaptations. Choosing fields with no-risk factors may reduce damage risk for all crops, including sensitive vegetable crops (e.g., potatoes).

### IPM targets

Assessing the risk of wireworm damage affords a solid basis for estimating the amount of farmland that can be left untreated each season without any risk of yield reduction.

In Italy, implementing IPM is likely to result in a maximum of 4 % of maize-cultivated land being treated with soil insecticides or insecticide-coated seeds.

### A look at the past

This 29-year survey clearly reveals that soil insecticides were used on a much wider area of maize crops than was necessary, and that by applying the risk factors outlined herein, soil-insecticide use can be restricted to fields where the probability of damage is considerable and the wireworm populations exceed the threshold (Furlan 2014). Over the past 30 years, most of Italy’s maize fields were treated with soil insecticides as seed dressing or in-furrow micro-granular application (Furlan and Kreutzweiser 2015), but this was cost-effective only for a very restricted number of fields. Therefore the significant environmental impact caused (van der Sluijs et al. 2015) was of no general benefit and most likely harmful for operators and other living organisms.

### A look at the future

The same principles may be applied to future pest management. Precise targets for IPM of soil pests in maize could be set everywhere (Fig. 2). For instance, in no-risk areas, soil insecticides or insecticide-coated seeds may need to be used on no more than 1 % of maize-cultivated land, and in areas where organic matter content is over 5 %, soil insecticides could be used on about 20 % of maize-cultivated land when the prevalent species is, for example, *Agriotes sordidus* (Fig. 2). For large areas with scattered-risk situations, IPM thresholds will be a balanced mean of the damage risk caused by various risk factors and the surface area of cultivated land where each risk factor occurs. This could be immediately applied to areas harbouring the species studied herein and to other areas shortly afterwards. In fact, local checks and adaptations should be assessed in regions where other species and/or conspicuous climatic differences occur, but the aforementioned IPM approach could be used since it is likely that the same main risk factors play a key role. Although Fig. 2 gives the main information for implementing IPM of wireworms in different European countries, a simple software (available on request) has been developed to make it easier and quicker to simulate combinations of risk factors in a range of areas. This would allow IPM to be extended to wherever the *Agriotes* species studied in this work are widespread, and probably also to wherever other Elateridae species occur, once accurate comparisons have been made. In order to facilitate IPM, risk insurance coverage may be extremely useful. Insurance may be taken out privately by associated farmers, or with the support of EU regulations (Reg. 2013/1305/EU). With risks below 1 %, a small amount of money per hectare (ten times less than soil-insecticide costs) would be enough to pay for damaged fields (Ferrari et al. 2015), including those damaged due to the failure of soil insecticides, the likelihood of which is high (Ferro and Furlan 2012; Saussure et al. 2015).
